# Cross-Protective Efficacy of Monovalent Live Influenza B Vaccines against Genetically Different Lineages of B/Victoria and B/Yamagata in Ferrets

**DOI:** 10.1155/2018/9695628

**Published:** 2018-08-30

**Authors:** Irina Kiseleva, Elena Krutikova, Ekaterina Stepanova, Svetlana Donina, Maria Pisareva, Vera Krivitskaya, Andrey Rekstin, Erin Grace Sparrow, Guido Torelli, Larisa Rudenko

**Affiliations:** ^1^Institute of Experimental Medicine, St Petersburg, Russia; ^2^World Health Organization, Geneva, Switzerland

## Abstract

**Background:**

Currently, two genetic lineages of influenza B virus, B/Victoria and B/Yamagata, are cocirculating in humans in various countries. This situation has raised a question regarding the possibility of cross-protection between B components of live attenuated influenza vaccine (LAIV) belonging to different lineages. This study aimed to assess in naïve ferrets the potential protective activity of monovalent B-LAIVs against challenge with homologous and heterologous wild-type (WT) influenza B viruses.

**Methods:**

Groups of seronegative female ferrets 5-6 months of age were given one dose of monovalent LAIV based on B/Victoria or B/Yamagata lineage virus. Ferrets were challenged 21 days later with B/Victoria or B/Yamagata WT virus. Ferrets were monitored closely for clinical signs and morbidity outcomes including febrile response, body weight loss, nasal symptoms, and level of activity one week prior to vaccination and for three days following vaccination/challenge. Nasal washes were collected three days after vaccination/challenge. Samples of lung tissue were taken three days after challenge. All samples were analyzed for the presence of challenge virus by culturing in embryonated chicken eggs and real-time polymerase chain reaction. Antibody response to vaccination was assessed by routine hemagglutination inhibition assay and microneutralization test.

**Results:**

Vaccination led to intensive production of specific neutralizing and antihemagglutinating antibodies to vaccine virus, protected ferrets from homologous challenge infection, and significantly reduced clinical signs and replication of homologous challenge virus. In contrast, cross-lineage serum antibodies were not detected. However, ferrets vaccinated with monovalent B-LAIV had a significantly lower level of heterologous challenge virus in the respiratory tract than those given challenge virus only.

**Conclusions:**

Monovalent B-LAIV has the potential to be cross-protective against infection with genetically different influenza lineages. Further studies are required to confirm this effect.

## 1. Introduction

Influenza is responsible for considerable morbidity and mortality, accounting for up to 5 millions of severe illness worldwide [1]. The unpredictable character of influenza epidemics and pandemics is a significant threat to human health.

Immunization with influenza vaccine is the primary measure for preventing influenza [2]. In recent years, interest in live attenuated influenza vaccine (LAIV) has significantly increased. The Global Action Plan for Influenza Vaccines gave attention to LAIVs for use in pandemics [3], because they stimulate a broad immune response [4–6]. Other benefits of live vaccines are intranasal administration; the possibility of producing large amounts of vaccine rapidly; and their ability to protect against antigenically drifted viruses [4, 6, 7].

The success of influenza vaccination depends significantly on the antigenic match between circulating strains and the strains included in the vaccine. In the past, there has been a mismatch between vaccine components and the most prevalent epidemic influenza viruses in a number of influenza seasons [8–11]. However, influenza vaccines, especially LAIV, may induce broad-spectrum and long-lasting immune responses and provide protection against drifted influenza viruses [4, 6, 12, 13].

The proportion of influenza B burden during influenza season can reach up to 80% depending on season and country [14]. Currently, two genetic lineages of influenza B virus—B/Victoria and B/Yamagata—are circulating among humans, with one or the other lineage being more prevalent in specific countries and regions. The frequent mismatch between the vaccine and circulating strains of influenza B viruses makes broadly effective influenza B vaccines an important public health need [11, 15, 16]. The current situation raises important questions regarding the possibility of cross-protection between B components of LAIV belonging to different genetic lineages.

Since the 1990s, some studies have indicated that vaccines containing B/Yamagata/16/88 may adequately protect adults against B/Victoria/2/87 infections [17, 18]. While influenza B causes disease in all age groups, children are most sensitive. However, in immunologically naïve children, vaccination with a B/Yamagata-like vaccine strain did not induce detectable hemagglutination-inhibiting or neutralizing antibody to B/Victoria-like viruses [19]. The absence of cross-reacting serum antibodies has also been confirmed in experiments on ferrets [20].

Nevertheless, experiments in mice demonstrated that IgA in nasal secretions may provide cross-protection against challenge infection with heterologous influenza B virus [21]. An* in vitro* study confirmed that virus-specific polyclonal CD8+ cytotoxic T-lymphocyte populations obtained from human leukocyte antigen (HLA) typed healthy study subjects cross-reacted with heterologous influenza B virus [22].

These data indicate a potential for cross-protection against antigenically distinct lineages of influenza B viruses through local and cellular immune response mechanisms.

The aim of this study was to assess in naïve ferrets the protective activity of monovalent B-LAIVs against challenge with homologous and heterologous wild-type (WT) influenza viruses belonging to genetically different lineages of B/Victoria and B/Yamagata.

## 2. Materials and Methods

### 2.1. Viruses

Vaccine candidates for seasonal LAIV, B/60/Brisbane/2008/83 (Victoria lineage), and B/60/Phuket/2013/26 (Yamagata lineage) are live, attenuated, cold-adapted (*ca*), temperature-sensitive (*ts*) reassortant influenza viruses. They were generated by the Institute of Experimental Medicine (St Petersburg, Russia) in embryonated chicken eggs by classical reassortment of B/USSR/60/69* ca/ts* master donor virus (MDV) with B/Brisbane/60/2008 (Victoria lineage) or B/Phuket/3073/2013 (Yamagata lineage) WT influenza B viruses obtained from the Centers for Disease Control and Prevention (CDC), Atlanta, USA. The vaccine viruses contain six gene segments encoding the internal proteins from the MDV and the hemagglutinin (HA) and neuraminidase (NA) proteins from the WT virus (6:2 genomic composition). B/Brisbane/60/2008 and B/Phuket/3073/2013 WT influenza B viruses were also used as challenge viruses.

All viruses were propagated in 10-11-day-old embryonated chicken eggs.

### 2.2. Ferrets (Mustela putorius furo)

Female ferrets aged 5-6 months were supplied by Scientific-Production Organization House of Pharmacy JSC (St Petersburg, Russia). They were prescreened by routine hemagglutination inhibition (HAI) test to ensure that they were negative to both circulating human influenza viruses and the viruses being tested. The animals were housed and the main procedures were carried out in accordance with European Union legislation [23]. At the end of the study, animals were euthanized with a combination of Zoletil and Xylazine. All procedures were approved by the local bioethical committee of the Institute of Preclinical Research Ltd (St Petersburg, Russia).

### 2.3. Study Design

**Figure 1:**
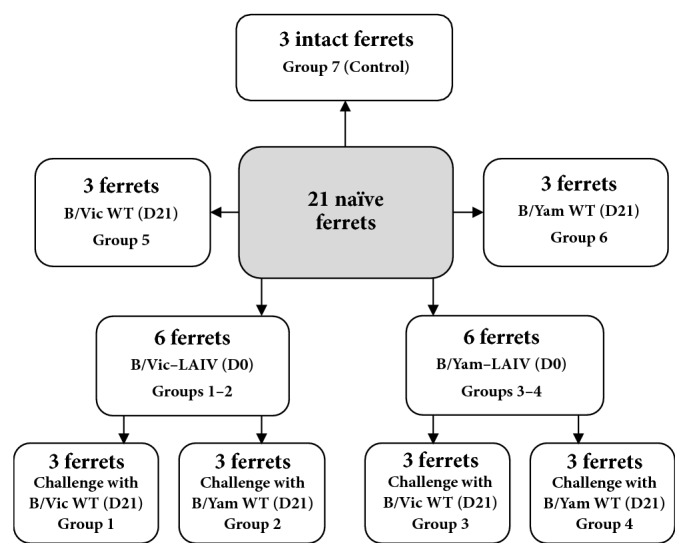
Study design.

The study population (21 naïve female ferrets 5-6 months of age) was used to determine the estimates of the potential of monovalent B-LAIV to be cross-protective against infection with genetically different influenza lineages. Two study groups of three ferrets each were immunized intranasally with a single dose of 7.0 log_10_⁡50% egg infectious dose (EID_50_/ml) of B/60/Brisbane/2008/83 (Victoria lineage) LAIV (B/Vic-LAIV) in a volume of 1 ml divided between the two nostrils, on study day 0, under inhalation anesthesia with isoflurane (groups 1 and 2). Another two study groups of three ferrets each were immunized intranasally with a single dose of 7.0 log_10_⁡EID_50_/ml of B/60/Phuket/2013/26 (Yamagata lineage) LAIV (B/Yam-LAIV) in a volume of 1 ml divided between the two nostrils, on study day 0, under inhalation anesthesia with isoflurane (groups 3 and 4). Three additional study groups of three ferrets each (groups 5, 6, and 7) were used as controls. Ferrets of groups 5 and 6 received no LAIV on study day 0; three weeks later they were inoculated with 6.0 log_10_⁡EID_50_/ml of B/Brisbane/60/2008 (Victoria lineage) or B/Phuket/3073/2013 (Yamagata lineage) WT virus in a volume of 1 ml (groups of control of challenge virus). Ferrets of group 7 did not receive either LAIV or WT virus (control of intact animals) (Figure 1). Three weeks after immunization (day 21), ferrets in groups 1, 4, and 5 were challenged with 6.0 log_10_⁡EID_50_/ml of B/Brisbane/60/2008 (Victoria lineage) WT virus in a volume of 1 ml. Ferrets in groups 2, 3, and 6 were challenged with 6.0 log_10_⁡EID_50_/ml of B/Phuket/3073/2013 (Yamagata lineage) WT virus in a volume of 1 ml. Group 7 ferrets were not challenged. Blood samples for serum preparation were collected on study days 0 and 21.

### 2.4. Clinical Signs and Morbidity Outcomes

Ferrets were monitored closely one week prior to inoculation and for three days following vaccination and challenge. Clinical signs were assessed; ferrets were evaluated for nasal symptoms and level of activity. Nasal symptoms were scored as follows: 1: nasal rattling could be heard or the ferret sneezed during transport from its cage to the evaluation area; 2: animals showed evidence of nasal discharge on their external nares; 3: animals exhibited mouth breathing; 0: none of these symptoms was seen.

Activity level was scored over a range of 0 to 3, depending on the extent to which the animal could be induced to play: 3: animal was fully playful; 2: animal responded to play overtures but did not initiate any play activity; 1: animal was alert but not at all playful; 0: animal was neither playful nor alert. Results are presented as an average score per group.

The body temperature of the animals was recorded using a digital thermometer prior to inoculation (day 0) and once a day on days 1-3 and 21-24. Body weight was also measured prior to inoculation (day 0) and on days 1-3 and 21-24 after inoculation. The temperature of 39.0°C was considered as a cut-off temperature.

### 2.5. Virus Replication in Ferrets' Airways

Nasal washes were collected three days after vaccination and challenge for virological analysis and real-time polymerase chain reaction (PCR). Samples of lung tissue were taken three days after challenge (day 24) and analyzed for the presence of challenge virus by titration in embryonated chicken eggs and real-time PCR.

#### 2.5.1. Vaccine Virus Isolation in Embryonated Chicken Eggs

Nasal wash specimens and lung tissue samples have been taken in accordance with [24] and were cultured in embryonated chicken eggs for detection of viral shedding. Nasal wash aliquots were inoculated in 10-11-day-old embryonated chicken eggs (Nazia poultry plant, St Petersburg, Russia) and incubated at 32°C for 72 hours. Eggs were subsequently chilled overnight before harvesting. The presence of influenza virus was detected by standard hemagglutination (HA) test with 1% chicken red blood cells [24].

#### 2.5.2. PCR-Based Virus Detection

To estimate influenza virus RNA level, a method of threshold cycle (Ct) comparison was used (determination of the number of PCR cycles necessary to achieve a given level of fluorescence). Nasal wash specimens were tested by PCR for the presence of influenza B virus RNA. RNA was extracted from the nasal washes using a RIBO-sorb reagent kit for RNA/DNA isolation (InterLabService, Central Research Institute of Epidemiology under Rospotrebnadzor, Moscow, Russia). Real-time PCR testing using SuperScript III Platinum One-step qRT-PCR System (Invitrogen) and primers and probes for influenza virus RNA amplification (CDC, Atlanta, USA) were used to detect virus RNA.

### 2.6. Antibody Response

The antibody response to vaccination was assessed by routine HAI and microneutralization (MN) tests. HAI was performed using a standard procedure [24] with chicken red blood cells, using 4 hemagglutinating units of influenza virus. Serum samples were pretreated with receptor destroying enzyme (RDE, Denka Seiken, Tokyo, Japan) to eliminate inhibitors of nonspecific hemagglutination when performing the HAI test. The MN test [24] was performed using a Madin-Darby canine kidney (MDCK) cell line after serum treatment with RDE. A fourfold or greater rise in antibody titer after vaccination was considered a reliable indicator of seroconversion.

### 2.7. Statistical Analysis

Data were analyzed with Statistica 10.0 (StatSoft, USA). The Shapiro-Wilk test was used for normality assessment. Differences between groups were analyzed using Student's* t*-test, Mann-Whitney* U* test, Median test, Kruskall-Wallis ANOVA by ranks with post hoc Dunnett test, and one-way ANOVA with post hoc Tukey test. Differences were considered significant at p ≤ 0.05. Data are shown as mean values (± standard error of the mean) or median (lower quartile-upper quartile).

## 3. Results

### 3.1. Clinical Observations after Vaccination

No clinically significant adverse events were observed in vaccinated ferrets. The immunized animals developed mild clinical signs, such as discharge from the nose, sneezing, and catarrhal symptoms, from day 1 onwards. No signs of fever were noticed. The activity of the vaccinated animals decreased slightly on days 1 and 2 after vaccination but returned to normal on D3 (average score of activity on D3 was 2.5-3.0) (Table 1). Changes in body weight among the vaccinated animals were not significantly different from those in the unvaccinated unchallenged group 7 of intact ferrets (data not shown). These data demonstrate the absence of vaccine side effects.

### 3.2. Clinical Observations after Challenge with WT Virus

All 18 ferrets challenged with WT virus survived the three-day follow-up period. The control animals (groups 5 and 6) showed more pronounced activity loss than the vaccinated ferrets. The general condition of the vaccinated animals after challenge was close to the normal physiological state. One dose of monovalent LAIV significantly reduced clinical signs in ferrets after challenge. While challenge of vaccinated ferrets led to some decreased activity, their activity was higher than that of unvaccinated challenged animals. The ferrets vaccinated with B/Phuket/3073/2013 (Yamagata lineage) and challenged with the same WT virus (group 3) had a statistically significantly higher level of activity than the challenged control animals (group 6) on day 24 (Kruskall-Wallis ANOVA, p<0.05, Dunnett post hoc test p=0.007) (Table 2).

Elevated body temperatures were detected in the control groups of ferrets as early as one day after inoculation with WT influenza B virus and persisted for up to two days. In contrast, vaccinated ferrets showed no signs of fever after challenge with WT virus (Table 2).

**Figure 2:**
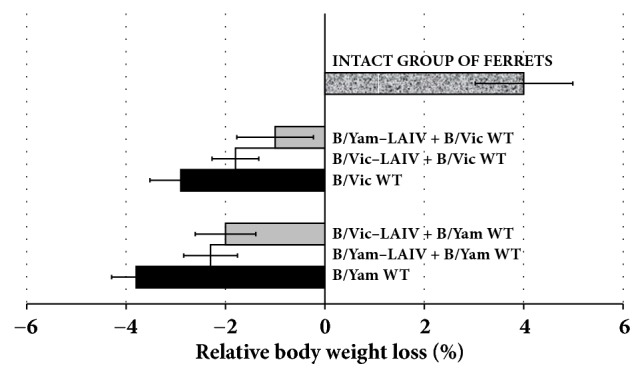
Percentage change in body weight from day 21 to day 24.

The control animals inoculated with B/Brisbane/60/2008 (Victoria lineage) WT virus (group 5) or B/Phuket/3073/2013 (Yamagata lineage) WT virus (group 6) showed a mean loss of body weight of 2.9% and 3.8%, respectively. Mean weight loss in the vaccinated animals after challenge infection was lower: 1.8%, 2.0%, 2.3%, and 1.0% for groups 1, 2, 3, and 4, respectively. The unchallenged unvaccinated group of ferrets (group 7) showed a 4% increase in body weight from day 21 to day 24 (Figure 2).

### 3.3. Vaccine Virus Replication

Replication of the vaccine viruses in the upper respiratory tract was assessed on day 3 after vaccination by titration of nasal washes in embryonated chicken eggs. The virus titers ranged from 3.7 to 3.9 log_10_⁡EID_50_/ml (Table 3).

The presence of genetic material of influenza B virus in the airways of the experimental and control groups was also evaluated using real-time PCR. The less virus there is in the samples, the greater the number of cycles needed to reach background fluorescence level. Thus, lower values of Ct indicate a higher concentration of influenza virus RNA in the sample. Ct values are shown in Table 4. On day 3 after vaccination, genetic material of vaccine viruses was found in all vaccinated ferrets.

### 3.4. Challenge Virus Replication

None of the vaccinated ferrets (groups 1-4) had detectable replication of either the homologous or the heterologous challenge virus (B/Brisbane/60/2008 or B/Phuket/3073/2013) in the lungs (lower limit of detection 1.5 log_10_⁡EID_50_/ml per gram of tissue).

In contrast, the control animals inoculated with WT virus showed average lung viral titers of 3.01 (B/Brisbane/60/2008) and 0.96 (B/Phuket/3073/2013) log_10_⁡EID_50_/ml per gram of tissue.

Challenge viruses were isolated from nasal washes of vaccinated animals, but the titers were statistically significantly lower than in the control ferrets (Mann-Whitney U, P < 0.05). In the vaccinated animals, mean titers ranged from 0.7 to 1.83 log_10_⁡EID_50_/ml, while in control groups 5 and 6 they were 5.53 and 3.7 log_10_⁡EID_50_/ml, respectively (Table 3).

These results were largely confirmed by real-time PCR assay. WT challenge virus was not detected in the lungs of ferrets in groups 1-3 (B/Vic-LAIV + B/Vic WT, B/Vic-LAIV + B/Yam WT, and B/Yam-LAIV + B/Yam WT). In contrast, genetic material of B/Vic WT challenge virus was found in the lungs of ferrets vaccinated with B/Yam-LAIV (Ct = 29.98 ± 0.95).

These results indicate that vaccination with monovalent B-LAIV may protect ferrets against challenge with type B viruses of different genetic lineages. In particular, vaccination inhibits replication of both homologous and heterologous challenge virus. However, vaccination with B/Vic-LAIV had a more pronounced effect on the replication of the heterologous challenge virus than vaccination with B/Yam-LAIV.

The lower virus replication in vaccinated animals was accompanied by correspondingly fewer clinical symptoms.

### 3.5. Antibody Responses

None of the ferrets had HAI antibody titers ≥ 1:5 to B/Brisbane/60/2008 (Victoria lineage) or B/Phuket/3073/2013 (Yamagata lineage) influenza viruses prior to B-LAIV vaccination. Twenty-one days after the vaccination, all ferrets presented a fourfold or greater increase in HAI antibody titers to the homologous WT challenge virus.

In the MN test, a fourfold or greater increased response to the homologous WT virus was detected in all vaccine recipients. Geometric mean titers (GMTs) of HAI and virus neutralizing antibodies were significantly higher on day 21 in vaccinated animals, but not in the control groups 5-7. In contrast, no cross-lineage antibody response was detected. None of the ferrets had HAI or MN antibody titers to the heterologous WT challenge virus (Table 5).

## 4. Discussion

Influenza B virus heterogeneity is driven by antigenic drift and lineage turnover [25]. In the 1970s, influenza B viruses diverged into two major antigenically distinct lineages, B/Victoria, and B/Yamagata [26]. Since then, there have been two parallel evolutionary pathways of influenza type B in the human population [9, 27].

Seasonal trivalent influenza vaccines include only one influenza B strain, while quadrivalent vaccines contain two B strains. For trivalent vaccines, a mismatch between the lineage of the influenza B vaccine virus and the circulating strain may result in reduced vaccine effectiveness and increased morbidity, especially among children [8, 10, 11]. Quadrivalent vaccines are increasingly being used to address this problem. However, an alternative approach could be to develop vaccines that induce cross-protection to antigenically distinct lineages of influenza B. Serum antibody response is the most commonly used measure of vaccine effectiveness [28]. Cross-reactivity between drifted influenza B variants has been demonstrated in experimental and clinical observations [11, 19]. However, most studies have found no evidence of cross-reacting serum HAI or neutralizing antibodies to influenza B virus of different lineage [19, 20, 29].

While the response to inactivated influenza vaccine is strain-specific, LAIV provides broad immunity against circulating strains, including heterotypic immunity [4, 12]. Local immunity stimulated by LAIV immunization may play a major role in preventing influenza [4]. Intranasal vaccination of mice with B/Victoria- and B/Yamagata lineage virus vaccines displayed cross-protective immunity, not only against homologous- but also heterologous-lineage virus infection. The efficacy of cross-protection correlated with the degree of induction of secretory IgA (S-IgA) antibodies. S-IgA antibodies induced by intranasal B virus inoculation most strongly protected mice against infection with a homologous virus or with drifted viruses within the homologous lineage; they offered weak protection against infection with heterologous-lineage viruses [21].

The virus-specific CD8^+^ T-cell-response also has an important role in preventing influenza infection [30]. It has been shown that cytotoxic T-lymphocyte responses directed to B/Victoria lineage viruses cross-react with B/Yamagata lineage viruses and vice versa [22].

In our experiments, one dose of monovalent B-LAIV led to intensive production of specific virus neutralizing and antihemagglutinating antibodies to homologous virus. In contrast, cross-lineage antibody response was not detected. However, serum antibody response is not an exclusive indicator of vaccine efficacy; different parts of the immune system are involved in resistance to influenza [4]. The assessment of respiratory symptoms, animal activity, and virological data demonstrated the effectiveness of vaccination and the cross-protectivity of influenza B-LAIVs. This cross-protection may be associated with local immunity and cell-mediated immune response.

The clinical signs of infection after homologous and heterologous challenge were significantly milder in vaccinated animals than in control groups; the general physiological state of the vaccinated animals was close to normal. In control groups, infection with wild-type influenza viruses of B/Yamagata or B/Victoria lineages was accompanied by nasal discharge on days 22-24, a decrease in activity and body weight, and a slight increase in body temperature. Interestingly, nasal discharge after infection with B/Yamagata strain was more pronounced than after infection with B/Victoria strain. This may be explained by the preferential binding of B/Yamagata strain to *α*-2,6-linkage glycan [31], which is common in the upper respiratory tract of ferrets [32].

Importantly, neither B/Vic WT nor B/Yam WT challenge viruses were detected in the lungs of vaccinated ferrets by culturing in eggs. In contrast, unvaccinated animals inoculated with B/Vic WT or B/Yam WT viruses showed average lung viral titers of 3.01 and 0.96 log_10_⁡EID_50_/ml per gram of tissue, respectively. This difference in the recovery of B/Vic and B/Yam WT viruses from the lungs of control animals could also be explained by the fact that B/Yamagata-like strains preferentially bind to *α*-2,6-linkage glycan, which is predominant in the upper respiratory tract, while Victoria-like strains bind to both *α*-2,3- and *α*-2,6-linkages [31].

Real-time PCR is more sensitive than virus isolation in developing chicken eggs [33]. Genetic material of influenza B/Vic challenge virus was detected by PCR in the lung tissue of animals vaccinated with B/Yamagata. However, PCR detects not only infective virus particles, but also incompletely packaged virus or fragmented RNA, which could produce false-positive results.

A previous ferret challenge study demonstrated protection only against homologous lineage B virus [20]. Ferrets vaccinated with B/Yamagata LAIV were challenged with B/Yamagata or B/Victoria lineage viruses. None of the ferrets challenged with B/Yamagata had challenge virus in their lungs, while all ferrets challenged with B/Victoria did. Unfortunately, this study did not look at cross-protection after B/Victoria immunization [20]. Our study also found genetic material of B/Victoria challenge virus in lungs of ferrets vaccinated with B/Yamagata LAIV. However, we demonstrated limited cross-protection against influenza B/Victoria lineage virus infection.

Surprisingly, cross-protection after B/Victoria vaccination against B/Yamagata challenge was stronger than vice versa. In [21] cross-reacting IgA titers after B/Yamagata infection of mice with B/Victoria challenge were slightly lower than a level of cross-reactive IgA after B/Victoria infection and subsequent B/Yamagata challenge. Clinical trials on young children vaccinated with trivalent inactivated influenza vaccine demonstrated that immunization with two doses of B/Yamagata-containing vaccine did not adequately prime children for response to subsequently immunization with B/Victoria lineage antigen [34]. At the same time, B/Victoria-containing vaccine strongly boosted serum antibody response to B/Yamagata virus. Additional studies are necessary to understand if it is a specific peculiarity of strains used or this is common for influenza B viruses belonging to different lineages.

In summary, intranasal vaccination with B/Vic-LAIV or B/Yam-LAIV conferred cross-protection not only against homologous- but also heterologous-lineage virus infection in ferrets. B-LAIV vaccination most strongly protects against infection with a homologous B virus. However, B/Vic-LAIV strongly protected ferrets against infection with B/Yam WT virus, while B/Yam-LAIV weakly protected against infection with B/Vic WT virus.

## 5. Conclusion

One dose of either B/60/Brisbane/2008/83 (Victoria lineage) or B/60/Phuket/2013 (Yamagata lineage) monovalent LAIV led to production of specific virus neutralizing and antihemagglutinating antibodies to vaccine virus, protected ferrets from homologous challenge infection, and reduced clinical signs and replication of homologous challenge virus. Vaccinated animals maintained a near-normal physiological state after challenge infection with WT virus. In contrast, unvaccinated ferrets challenged with WT virus had virus replication in the upper and lower respiratory tract; symptoms of respiratory disease were observed as well as a decrease in overall activity.

A cross-lineage antibody response was not detected. However, our results show that both monovalent B-LAIVs may have the potential to protect against infection with genetically different influenza B lineages. This indicates that other immune mechanisms (for instance, local and/or cellular immune response) may be involved. Vaccination with B/Victoria LAIV had a more pronounced effect on the replication of the heterologous B/Yamagata challenge virus than vaccination with B/Yamagata LAIV had on B/Victoria challenge virus. Further studies should be conducted to confirm this effect.

## Figures and Tables

**Table 1:** Clinical observations in ferrets after vaccination with monovalent B-LAIV.

Group	B/Vic LAIV (groups 1-2)				B/Yam LAIV (groups 3-4)				Intact animals (group 7)			
Day	Day 0	Day 1	Day 2	Day 3	Day 0	Day 1	Day 2	Day 3	Day 0	Day 1	Day 2	Day 3
Body temperature (°C)	38.4	38.3	38.7	38.8	38.7	38.8	38.9	38.8	38.5	38.7	38.6	38.7
Activity [group Median (Q1-Q3)]	3.0 (3.0-3.0)	2.5 (1.0-3.0)	2.5 (1.0-3.0)	3.0 (3.0-3.0)	3.0 (3.0-3.0)	1.5 (0.0-2.0)	2.0 (2.0-2.0)	2.5 (2.0-3.0)	3.0 (3.0-3.0)	3.0 (3.0-3.0)	3.0 (3.0-3.0)	3.0 (3.0-3.0)
Respiratory symptoms [group Median (Q1-Q3)]	0.0 (0.0-0.0)	0.5^1^ (0.0-1.0)	0.5^1^ (0.0-1.0)	0.5^1^ (0.0-1.0)	0.0 (0.0-0.0)	1.0^1^ (1.0-1.0)	1.0^1^ (1.0-1.0)	1.0^1^ (0.0-1.0)	0.0 (0.0-0.0)	0.0 (0.0-0.0)	0.0 (0.0-0.0)	0.0 (0.0-0.0)

Abbreviations are used here and in Tables 2–5 and Figures 1 and 2: B/Yam LAIV, monovalent LAIV based on B/60/Phuket/2013/26 (B/Yamagata lineage); B/Vic LAIV, monovalent LAIV based on B/60/Brisbane/2008/83 (B/Victoria lineage); B/Yam WT, B/Phuket/3073/2013 wild type virus (B/Yamagata lineage); B/Vic WT, B/Brisbane/60/2008 wild-type virus (B/Victoria lineage). Statistically significant differences: ^1^median test between groups, p≤0.05, post hoc Dunnett test, p≤0.05 (versus intact group).

**Table 2:** Clinical observations in ferrets after challenge with B/Brisbane/60/2008 (B/Vic) or B/Phuket/3073/2013 (B/Yam) wild-type influenza virus.

After challenge with B/Vic WT virus	Body temperature (°C)				Activity (group average)				Respiratory symptoms (group average)			
	Day 21	Day 22	Day 23	Day 24	Day 21	Day 22	Day 23	Day 24	Day 21	Day 22	Day 23	Day 24
B/Vic LAIV (group 1)	38.7	38.4	38.8	38.5	3.0	2.0	2.3	2.7	0.0	0.0	0.0	0.0
B/Yam LAIV (group 4)	38.8	38.4	38.5	38.2	3.0	2.3	2.3	2.3	0.0	0.7	1.0^1^	0.3
Unvaccinated control (group 5)	38.9	39.2	39.0	38.5	3.0	1.0^1^	1.7^1^	2.3	0.0	0.7	0.7	0.3
Intact animals (group 7)	38.5	38.4	38.8	38.4	3.0	3.0	3.0	3.0	0.0	0.0	0.0	0.0

After challenge with B/Yam WT virus	Body temperature (°C)				Activity (group average)				Respiratory symptoms (group average)			
	Day 21	Day 22	Day 23	Day 24	Day 21	Day 22	Day 23	Day 24	Day 21	Day 22	Day 23	Day 24

B/Yam LAIV (group 3)	38.7	38.6	39.0	38.6	3.0	2.3	2.3	3.0^2^	0.0	0.7	0.7	0.0
B/Vic LAIV (group 2)	38.7	38.3	38.2	38.7	3.0	1.7^1^	1.7^1^	2.3	0.0	0.7	0.3	0.0
Unvaccinated control (group 6)	38.8	39.1	39.2	38.8	3.0	1.3^1^	1.7^1^	2.0^1^	0.0	1.0^1^	1.0^1^	0.7
Intact animals (group 7)	38.5	38.4	38.8	38.4	3.0	3.0	3.0	3.0	0.0	0.0	0.0	0.0

Statistically significant differences: ^1^Dunnett test, p≤0.05 (versus intact group); ^2^Dunnett test, p≤0.05 (vaccinated animals versus unvaccinated control).

**Table 3:** Virus replication in lungs and upper respiratory tract of ferrets (virus isolation in embryonated eggs).

Group	Vaccination		Challenge		
	Vaccine administered	Vaccine virus titer in nasal washes, day 3 (log_10_⁡EID_50_/ml)	WT challenge virus given, day 21	WT challenge virus titer in nasal washes, day 24 (log_10_⁡EID_50_/ml)	WT challenge virus titer in lung tissue, day 24 (log_10_⁡EID_50_/ml per g)
1	B/Vic LAIV	3.88 ± 0.119	B/Vic WT virus	0.90 ± 0.900	< 1.5
2	B/Vic LAIV		B/Yam WT virus	1.67 ± 0.953	< 1.5
3	B/Yam LAIV	3.68 ± 0.120	B/Yam WT virus	0.70 ± 0.700	< 1.5
4	B/Yam LAIV		B/Vic WT virus	1.83 ± 0.970	< 1.5
5	None	n.d.^1^	B/Vic WT virus	5.53 ± 0.167	3.01 ± 0.200
6	None	n.d.	B/Yam WT virus	3.70 ± 0.764	0.96 ± 0.957
7	None	< 1.5^2^	None	< 1.5	< 1.5

^1^n.d.: not determined. ^2^Estimated threshold limit value.

**Table 4:** Virus replication in lungs and upper respiratory tract of ferrets, as measured by real-time PCR.

Group	Vaccination		Challenge		
	Vaccine administered	Ct,^1^ day 3	WT challenge virus given, day 21	Ct in nasal washes, day 24	Ct in lung tissue, day 24
1	B/Vic LAIV	11.41 ± 0.611^2^	B/Vic WT virus	31.34 ± 4.663	negative
2	B/Vic LAIV		B/Yam WT virus	24.69 ± 1.462	negative
3	B/Yam LAIV	15.04 ± 0.806	B/Yam WT virus	negative	negative
4	B/Yam LAIV		B/Vic WT virus	19.46 ± 4.105	29.98 ± 0.950
5	None	n.d.^3^	B/Vic WT virus	14.92 ± 2.142	29.18 ± 0.566
6	None	n.d.	B/Yam WT virus	17.89 ± 0.552	27.86 ± 0.167
7	None	negative	None	negative	negative

^1^Ct: threshold cycle, the number of PCR cycles necessary to achieve a given level of fluorescence.

^2^ Mean value ± standard error of mean. ^3^n.d.: not determined.

**Table 5:** Antibody response to influenza B viruses.

Vaccine administered (groups)	Antigen					
	B/Vic WT (GMT)			B/Yam WT (GMT)		
	Day 0	Day 21	Fold increase	Day 0	Day 21	Fold increase
**HAI test**						

B/Vic LAIV (groups 1-2)	5.0	36.0	7.2	5.0	5.0	1.0

B/Yam LAIV (groups 3-4)	5.0	5.0	1.0	5.0	90.0	18.0

Groups 5-7 (controls)	5.0	5.0	1.0	5.0	5.0	1.0

**MN test**						

B/Vic LAIV (groups 1-2)	5.0	359.2	71.8	5.0	5.0	1.0

B/Yam LAIV (groups 3-4)	5.0	5.0	1.0	5.0	226.3	45.3

Groups 5-7 (controls)	5.0	5.0	1.0	5.0	5.0	1.0

## Data Availability

The results used to support the findings of this study are included within the article.

## References

[1] Influenza (Seasonal): Fact Sheet N°211. January 2018. World Health Organization. http://www.who.int/mediacentre/factsheets/fs211/en/.

[2] The ten years of the Global Action Plan for Influenza Vaccines. Report to the Director-General from the GAP Advisory group. Geneva: World Health Organization. http://www.who.int/influenza/GAP_AG_report_to_WHO_DG.pdf.

[3] Global influenza pandemic action plan to increase vaccine supply. Geneva: World Health Organization; 2006 (WHO/IVB/06.13). http://whqlibdoc.who.int/hq/2006/WHO_IVB_06.13_eng.pdf.

[4] Cox R. J., Brokstad K. A., Ogra P. (2004). Influenza virus: immunity and vaccination strategies. Comparison of the immune response to inactivated and live, attenuated influenza vaccines. *Scandinavian Journal of Immunology*.

[5] Hoft D. F., Babusis E., Worku S. (2011). Live and inactivated influenza vaccines induce similar humoral responses, but only live vaccines induce diverse T-cell responses in young children. *The Journal of Infectious Diseases*.

[6] Tamura S.-I., Tanimoto T., Kurata T. (2005). Mechanisms of broad cross-protection provided by influenza virus infection and their application to vaccines. *Japanese Journal of Infectious Diseases*.

[7] Nichol K. L., Treanor J. J. (2006). Vaccines for seasonal and pandemic influenza. *The Journal of Infectious Diseases*.

[8] Ambrose C. S., Levin M. J. (2012). The rationale for quadrivalent influenza vaccines. *Human Vaccines & Immunotherapeutics*.

[9] Centers for Disease Control and Prevention (2009). Update: influenza activity - United States, September 28, 2008-April 4, 2009, and composition of the 2009-10 influenza vaccine. *MMWR*.

[10] Centers for Disease Control and Prevention (2013). Prevention and control of seasonal influenza with vaccines. Recommendations of the Advisory Committee on Immunization Practices -United States, 2013-2014. *MMWR*.

[11] Trucchi C., Alicino C., Orsi A. (2017). Fifteen years of epidemiologic, virologic and syndromic influenza surveillance: A focus on type B virus and the effects of vaccine mismatch in Liguria region, Italy. *Human Vaccines & Immunotherapeutics*.

[12] Treanor J. J., Kotloff K., Betts R. F. (1999). Evaluation of trivalent, live, cold-adapted (CAIV-T) and inactivated (TIV) influenza vaccines in prevention of virus infection and illness following challenge of adults with wild-type influenza A (H1N1), A (H3N2), and B viruses. *Vaccine*.

[13] El-Madhun A. S., Cox R. J., Søreide A., Olofsson J., Haaheim L. R. (1998). Systemic and mucosal immune responses in young children and adults after parenteral influenza vaccination. *The Journal of Infectious Diseases*.

[14] Tafalla M., Buijssen M., Geets R., Noordegraaf-Schouten M. V. (2016). A comprehensive review of the epidemiology and disease burden of Influenza B in 9 European countries. *Human Vaccines & Immunotherapeutics*.

[15] Mosnier A., Caini S., Daviaud I. (2015). Ten influenza seasons in France: Distribution and timing of influenza A and B circulation, 2003-2013. *BMC Infectious Diseases*.

[16] Heikkinen T., Ikonen N., Ziegler T. (2014). Impact of influenza B lineage-level mismatch between trivalent seasonal influenza vaccines and circulating viruses, 1999-2012. *Clinical Infectious Diseases*.

[17] Levandowski R. A., Gross P. A., Weksler M., Station E., Williams M. S., Bonelli J. (1991). Cross-reactive antibodies induced by a monovalent influenza B virus vaccine. *Journal of Clinical Microbiology*.

[18] Camilloni B., Neri M., Lepri E., Iorio A. M. (2009). Cross-reactive antibodies in middle-aged and elderly volunteers after MF59-adjuvanted subunit trivalent influenza vaccine against B viruses of the B/Victoria or B/Yamagata lineages. *Vaccine*.

[19] Shaw M. W., Xu X., Li Y. (2002). Reappearance and global spread of variants of influenza B/Victoria/2/87 lineage viruses in the 2000-2001 and 2001-2002 seasons. *Virology*.

[20] Belshe R. B., Coelingh K., Ambrose C. S., Woo J. C., Wu X. (2010). Efficacy of live attenuated influenza vaccine in children against influenza B viruses by lineage and antigenic similarity. *Vaccine*.

[21] Asahi-Ozaki Y., Yoshikawa T., Iwakura Y. (2004). Secretory IgA antibodies provide cross-protection against infection with different strains of influenza B virus. *Journal of Medical Virology*.

[22] van de Sandt C. E., Dou Y., Trierum S. E. V. (2015). Influenza B virus-specific CD8+ T-lymphocytes strongly cross-react with viruses of the opposing influenza B lineage. *Journal of General Virology*.

[23] Directive 2010/63/EU of the European Parliament and of the Council of September 22, 2010, on the protection of animals used for scientific purposes. http://eur-lex.europa.eu/legal-content/EN/TXT/?uri=celex%3A32010L0063.

[24] WHO manual on animal influenza diagnosis and surveillance. Manila: WHO Regional Office for the Western Pacific. http://www.wpro.who.int/emerging_diseases/documents/docs/manualonanimalaidiagnosisandsurveillance.pdf.

[25] Chen R., Holmes E. C. (2008). The evolutionary dynamics of human influenza B virus. *Journal of Molecular Evolution*.

[26] Yang J., Huang Y., Chang F. (2012). Phylogenetic and evolutionary history of influenza B viruses, which caused a large epidemic in 2011–2012, Taiwan. *PLoS ONE*.

[27] Rota P. A., Wallis T. R., Harmon M. W., Rota J. S., Kendal A. P., Nerome K. (1990). Cocirculation of two distinct evolutionary lineages of influenza type B virus since 1983. *Virology*.

[28] Cox N. J., Subbarao K. (1999). Influenza. *The Lancet*.

[29] Levandowski R. A., Regnery H. L., Staton E., Burgess B. G., Williams M. S., Groothuis J. R. (1991). Antibody responses to influenza B viruses in immunologically unprimed children. *Pediatrics*.

[30] Sridhar S., Begom S., Bermingham A. (2013). Cellular immune correlates of protection against symptomatic pandemic influenza. *Nature Medicine*.

[31] Wang Y.-F., Chang C.-F., Chi C.-Y., Wang H.-C., Wang J.-R., Su I.-J. (2012). Characterization of glycan binding specificities of influenza B viruses with correlation with hemagglutinin genotypes and clinical features. *Journal of Medical Virology*.

[32] Belser J. A., Katz J. M., Tumpey T. M. (2011). The ferret as a model organism to study influenza A virus infection. *Disease Models & Mechanisms*.

[33] Lee C.-W., Suarez D. L. (2004). Application of real-time RT-PCR for the quantitation and competitive replication study of H5 and H7 subtype avian influenza virus. *Journal of Virological Methods*.

[34] Skowronski D. M., Hottes T. S., De Serres G. (2011). Influenza B/Victoria antigen induces strong recall of B/Yamagata but lower B/Victoria response in children primed with two doses of b/Yamagata. *The Pediatric Infectious Disease Journal*.

